# Bovine β-Casein Peptide YPFPGPIH Regulates Inflammation and Macrophage Activity via TLR/NF-κB/MAPK Signaling

**DOI:** 10.3390/foods14203572

**Published:** 2025-10-20

**Authors:** Junpeng Zhang, Xinyu Zhang, Guangqing Mu, Xiaomeng Wu, Jianping Wu

**Affiliations:** 1 School of Food Science and Technology, Dalian Polytechnic University, Dalian 116034, China; zhangjunpeng950808@gmail.com (J.Z.); 17866589682@163.com (X.Z.); wuxiaomeng0812@163.com (X.W.); 2 Department of Agricultural, Food and Nutritional Science, University of Alberta, Edmonton, AB T6G 2P5, Canada; jwu3@ualberta.com

**Keywords:** bovine β-casein derived peptide, immunomodulatory, NF-κB/MAPK signaling

## Abstract

Food-derived bioactive peptides are known to possess immunomodulatory properties, although their molecular mechanisms remain incompletely characterized. In this study, we investigated the immunoregulatory effects and underlying mechanisms of YPFPGPIH, a peptide derived from bovine β-casein, using the RAW264.7 macrophage model. Our results demonstrate that YPFPGPIH enhanced macrophage proliferation and phagocytosis in a dose-dependent manner and promoted chemotactic migration through the upregulation of monocyte chemoattractant proteins MCP-1 and MCP-3. Under lipopolysaccharide (LPS)-induced inflammatory conditions, YPFPGPIH significantly reduced the levels of pro-inflammatory mediators, including interleukin-1β (IL-1β), tumor necrosis factor-α (TNF-α), and nitric oxide (NO), while increasing the production of the anti-inflammatory cytokine interleukin-10 (IL-10), thereby reestablishing cytokine balance. Mechanistic studies revealed that YPFPGPIH inhibited LPS-induced activation of the NF-κB and MAPK pathways, as indicated by reduced nuclear translocation of p65 and decreased phosphorylation of ERK, JNK, and p38. Molecular docking analysis indicated strong binding affinities between YPFPGPIH and Toll-like receptors TLR2 and TLR4, suggesting the involvement of TLR-mediated signaling. Notably, YPFPGPIH downregulated inducible nitric oxide synthase (iNOS) expression and upregulated chemokine mRNA levels, reflecting its dual role in modulating inflammatory and migratory responses. These findings highlight YPFPGPIH as a multifunctional immunomodulatory peptide that fine-tunes macrophage activity through crosstalk between TLR, NF-κB, and MAPK signaling pathways. This study provides new insights for developing peptide-based therapeutics and functional foods aimed at managing inflammatory diseases.

## 1. Introduction

The immune system is the body’s primary defense mechanism, protecting against microbial infections and maintaining internal balance. Due to this essential role, it remains a major focus of biological research [1]. Food-derived bioactive peptides have gained significant attention in immunomodulation studies. They offer advantages such as low toxicity, high biocompatibility, and diverse physiological activities [2]. Unlike synthetic drugs, these peptides are safer and can help regulate immune function through dietary intake [3]. This makes them promising candidates for developing functional foods and novel immunomodulatory agents [4]. Milk protein hydrolysates, in particular, are rich in bioactive peptides with potent immunomodulatory properties. These peptides help maintain immune homeostasis, reduce inflammation, and support tissue repair by modulating both innate and adaptive immune responses [5].

The innate immune system depends on macrophages as its primary defenders. The functional state of these cells determines the effectiveness of immune responses [6]. Growing evidence shows that dietary bioactive peptides can precisely reprogram macrophage activity through multi-target modulation [7]. This includes influencing polarization phenotypes, phagocytic ability, and cytokine networks, ultimately reshaping the immune environment [8]. For example, the β-casein-derived peptide BCCY-1 activates the TLR4/NF-κB pathway to stimulate IL-10 production, establishing an anti-inflammatory feedback loop [9]. Similarly, silkworm pupa peptide (SPP) suppresses NF-κB, MAPK, and PI3K signaling, reducing expression of iNOS and COX-2 and thereby disrupting inflammatory cascades [10]. These findings indicate that peptides may enable precise immune cell programming through cooperative multi-target networks. Chemokine-directed cell migration is another essential aspect of inflammation. For instance, MCP-1/CCL2 and MCP-3/CCL7 guide monocytes and macrophages to inflamed tissues by binding to CCR2 [11]. Peptides can optimize such ligand–receptor interactions; one CXCL12-mimetic peptide promotes CXCR4 dimerization, improving mesenchymal stem cell homing [12]. This offers a promising model for designing migration-targeted therapies. At the molecular level, Toll-like receptors (TLRs) are central to peptide-mediated immunomodulation [13]. Activation of TLR2/4 by PAMPs or DAMPs triggers NF-κB and MAPK pathways, directly influencing inflammatory gene expression [14]. Notably, the peptide AMKPWIQPK interacts with both TLR2 and TLR4 on macrophages, enhancing immunoregulatory activity via MAPK/NF-κB signaling [15]. This suggests that peptide–TLR interactions may represent a common mechanism for tuning immune networks. Despite these advances, critical questions remain. Most studies focus on single pathways, lacking a multidimensional view of dynamic immune balance. It is still unclear how peptides coordinate pro- and anti-inflammatory signals to maintain immune homeostasis. The connection between chemokine-driven recruitment and TLR signaling also remains unexplored. In previous work, we obtained the peptide YPFPGPIH by simulating infant gastrointestinal digestion and found that its parent casein protein exerted immunoregulatory effects in immunosuppressed mice. To bridge these knowledge gaps, this study focuses on YPFPGPIH—a peptide derived from bovine β-casein—and systematically investigates its immunomodulatory functions and underlying mechanisms.

In this study, we used RAW264.7 macrophages to examine key immune cellular processes. Cell proliferation was measured with the CCK-8 assay, and flow cytometry was applied to analyze cell cycle distribution. Macrophage migration was evaluated using Transwell assays, while cytokine and chemokine levels were quantified via ELISA and quantitative PCR. Together, these approaches revealed the multifaceted effects of YPFPGPIH on macrophage function. To explore the underlying mechanisms, we performed computational docking to simulate peptide interactions with Toll-like receptors (TLRs). We also used immunofluorescence microscopy to track the intracellular localization of signaling molecules, particularly NF-κB nuclear translocation and MAPK pathway activation. This integrated strategy helped clarify how YPFPGPIH–TLR binding influences downstream signaling events. Our findings expand the potential applications of milk-derived proteins and provide a foundation for designing functional foods with immunomodulatory benefits.

## 2. Materials and Methods

### 2.1. Materials

The peptide YPFPGPIH, derived from bovine β-casein, was synthesized by DG Peptides Co., Ltd. (Hangzhou, China). The Dulbecco’s Modified Eagle Medium (DMEM), 0.5% Trypsin-EDTA, penicillin/streptomycin solution, and fetal bovine serum (FBS) were sourced from Meilun Biotechnology Co., Ltd. (Shanghai, China). ELISA kits for mouse MCP-1, MCP-3, IL-1β, IL-10, and TNF-α were acquired from Lengton Biotechnology Co., Ltd. (Shanghai, China). Lipopolysaccharides (LPS) were obtained from Macklin Biochemical Technology Inc. Co., Ltd. (Shanghai, China). The total RNA extraction kit for cells and the qPCR kit were purchased from Seven Biotechnology Co., Ltd. (Beijing, China). All other reagents were sourced from Beyotime Biotechnology Co., Ltd. (Shanghai, China).

### 2.2. Cell Proliferation, Cycle, and Migration Assays

The RAW 264.7 cell line, originating from murine macrophages, was obtained from the Shanghai Institute of Cell Biology (Shanghai, China). These cells were cultured in DMEM supplemented with 20% FBS and 1% penicillin–streptomycin antibiotic solution. The cells were cultured under conditions of 37 °C and 5% CO_2_ [16]. Cell viability was determined using the CCK-8 assay, according to Wang et al. [17]. The cells were cultured in varying polypeptide concentrations under standard conditions of 37 °C and 5% CO_2_. Basal medium was used as the negative control, while LPS at a concentration of 1 µg/mL served as the positive control in the experiment. Cells were seeded at a density of 5000 per well and incubated with peptide solutions at concentrations of 25 µM, 50 µM, and 100 µM for 24 h. Following incubation, 10 μL of CCK-8 solution was added to each well and further incubated at 37 °C for an additional 2 h. The optical density of the samples was then measured at 450 nm. Raw 264.7 cells were incubated for 24 h with polypeptides at varying concentrations: 25 µM, 50 µM, and 100 µM. Following fixation in 70% ethanol for 12 h, the cellular specimens were labeled with propidium iodide (PI) for 15 min under ambient conditions. Subsequently, the prepared samples were analyzed using a flow cytometry system from Beckman Coulter (Shanghai, China). Quantitative analysis of the cell cycle distribution was conducted using FlowJo software (v10.8.1) [18]. We conducted cell migration assays employing Transwell chambers. Culture supernatants, exposed to varying polypeptide concentrations (25 µM, 50 µM, and 100 µM), were introduced into the lower compartment of the chamber. Untreated culture supernatant and complete medium served as controls. A cell suspension with a density of 5 × 10^5^ cells/mL was placed into each well of the upper chamber. The assay was maintained at 37 °C within a 5% CO_2_ atmosphere for 24 h. After incubation, the membrane underwent sequential processing, including thorough washing, fixation with 4% paraformaldehyde, and staining with a 1% crystal violet solution. Subsequently, we counted the cells that had migrated and adhered to the underside of the membrane via microscopic examination [19].

### 2.3. Macrophage Phagocytosis

RAW 264.7 cells were cultured in 96-well plates at a density of 1 × 10^4^ cells/mL for 24 h. Following the incubation period, the cells were exposed to varying concentrations of polypeptide solutions. After 24 h of exposure, the culture medium was discarded, and each well was rinsed twice with PBS. Subsequently, 220 μL of a neutral red staining solution, prepared by diluting neutral red with PBS at a 1:10 ratio, was added to each well. The plates were then incubated for 2 h. The staining solution was removed, and the cells were washed twice with PBS. Thereafter, 200 μL of a cell lysis buffer, composed of a 1:1 mixture of acetic acid and ethanol, was added. The plates were incubated overnight. The phagocytosis rate (%) was calculated as follows [20]: Phagocytosis rate (%) = [(OD treatment − OD control)/(OD control − OD control)] × 100.

### 2.4. Immune Factor

RAW264.7 cells were seeded in 12-well plates at a concentration of 2 × 10^5^ cells per milliliter and maintained in culture for one day. Subsequently, the cells were exposed to lipopolysaccharide (LPS) at a dose of 1 µg/mL for another 24 h period. Next, the RAW 264.7 cells were treated with various concentrations of polypeptides for an additional 24 h. Finally, the appropriate kits were used to measure the levels of NO, IL-1β, IL-10, and TNF-α in the cell culture supernatant, while the iNOS in the cells was determined using an ELISA kit. RAW 264.7 cells were seeded in 12-well plates at a concentration of 2 × 10^5^ cells per milliliter and maintained in culture for one day. After the initial culture period, the cells were incubated with different peptide concentrations for an additional 24 h. Finally, the levels of chemokines MCP-1 and MCP-3 in the cell culture supernatants were quantified using the respective kits [21].

### 2.5. Relative Expression of Immune Factor mRNA

Following the treatment protocol’s completion, cellular samples were collected and washed with cold phosphate-buffered saline (PBS) to remove any residual therapeutic agents. Next, RNA was extracted from the treated cells using an RNA extraction kit. Gene expression levels were evaluated by quantitative reverse transcription polymerase chain reaction (qRT-PCR) with the ViiA 7 real-time PCR system (MA, USA). The expression primers for various immune factors can be found in Table 1.

### 2.6. Molecular Docking

We utilized Pymol and AutoDock to analyze the molecular interactions between YPFPGPIH and Toll-like receptors. Crystal structures of TLR2 [22] (entry code: 1FYW) and TLR4/MyDD [23] (entry code: 5IJD) were obtained from the Protein Data Bank. Water molecules were removed from the structures 1FYW and 55IJD before analysis. Docking parameters for 1FYW were set as follows: center_x = −2.699, center_y = 93.769, center_z = 22.546, size_x = 27.75, size_y = 30.0, size_z = 22.5. For 5IJD, the parameters were as follows: center_x = −9.452, center_y = 10.855, center_z = −0.191, size_x = 39.75, size_y = 25.5, size_z = 45.75. The visualization and analysis were performed with PyMOL 1.5 and AutoDock Vina software (v1.5.6).

### 2.7. Immunofluorescence Staining

RAW264.7 cells were cultivated in culture flasks until they reached approximately 80% confluence. First, we treated the cells with trypsin and seeded them into 12-well plates, dispensing 1 mL of cell suspension with a concentration of 5 × 10^4^ cells/mL per well. Following three PBS washes, we administered varying concentrations of peptide solutions to the cells. We maintained the cells in a 37 °C incubator with 5% CO_2_ for 24 h. After incubation, we removed the culture medium and performed a single PBS wash. We added 1 mL of cell tissue fixative to each well and allowed it to stand for 20 min. We washed the cells three times with PBS, with each wash lasting 5 min. Next, we added 1 mL of 0.1% Tinton to each well and incubate at room temperature for 1 h. Following this, we added 1 mL of blocking solution (BSA) to each well and left it at room temperature for another hour. Subsequently, we introduced 300 μL of the selected antibody (p65, JNK, ERK, or p38) into each well and maintain the incubation at 4 °C throughout the night. Following the retrieval of the primary antibody, we performed three consecutive washes using 1 mL of PBST, ensuring each washing step persisted for 10 min. Subsequently, we administered the secondary antibody and conducted the incubation in a light-protected environment for a duration of 1 h. After recovering the secondary antibody, we washed the cells twice with 1 mL of PBS, aspirating immediately after each addition. Finally, we added 50 μL of mounting solution (containing DAPI) to each well and observed the cells using an inverted fluorescence microscope [24].

### 2.8. Statistical Analysis

Data represent mean ± SD of three independent experiments, each performed in triplicate. Significance was determined by one-way ANOVA followed by Tukey’s post hoc test (*p* < 0.05). Least square means were derived using a general linear model (GLM) in GraphPad Prism 8.0.

## 3. Results and Discussion

### 3.1. Effects of YPFPGPIH on Macrophage Proliferation

**Figure 1:**
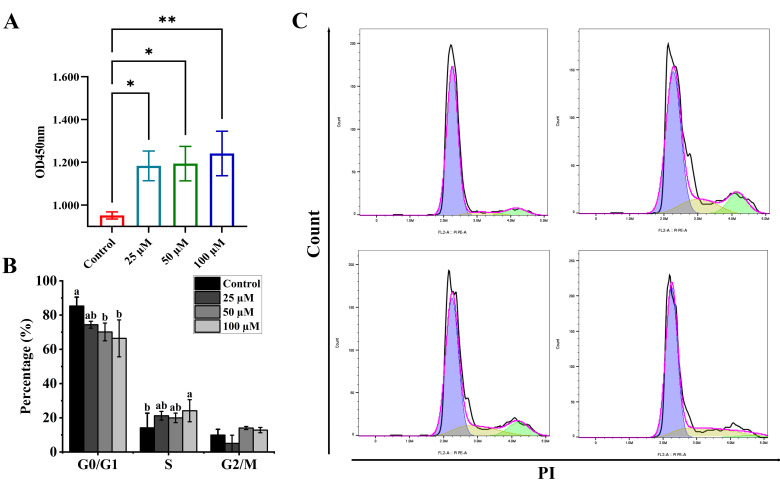
The effect of YPFPGPIH on RAW264.7 cells. (**A**) RAW264.7 cells were exposed to YPFPGPIH at concentrations of 0, 25, 50, and 100 µM for 24 h. Following this, cell proliferation was measured using the CCK-8 assay. Statistical analysis was conducted relative to the untreated control (0 µM), with significance denoted by * *p* < 0.05 and ** *p* < 0.01. (**B**,**C**) Flow cytometry was used to analyze the cell cycle distribution of RAW264.7 cells treated with YPFPGPIH at concentrations of 0, 25, 50, and 100 µM for 24 h. The percentages of cells in the G0/G1, S, and G2/M phases of the cell cycle are presented in (**B**), with a–b denoting different levels of significance.

The RAW264.7 cell line, derived from Abelson leukemia virus-transformed BALB/c mouse macrophages, retains essential features of primary macrophages—including TLR2/4 expression, phagocytic activity, and cytokine response to LPS stimulation [16,25]. These properties make it a suitable model for investigating innate immune mechanisms targeted by YPFPGPIH. To assess the effect of YPFPGPIH on macrophage proliferation, RAW264.7 cells were treated with various concentrations of the peptide for 24 h. As shown in Figure 1A, YPFPGPIH significantly enhanced cell proliferation (*p* < 0.05). Macrophage proliferation is known to be context-dependent and can be triggered during infection or inflammation [26,27]. Similarly, Li et al. reported that the casein hydrolysate SPAQILQW promotes macrophage proliferation [28]. Our results indicate that YPFPGPIH exerts a comparable effect. Cell proliferation is governed by progression through the cell cycle—G1, S, G2, and M phases [29]. The G1 phase supports growth and biomolecule accumulation, the S phase involves DNA replication, the G2 phase prepares for division, and the M phase culminates in mitosis [30]. These transitions are driven by cyclins and cyclin-dependent kinases (CDKs), which phosphorylate key substrates to advance the cycle [25]. We observed that YPFPGPIH treatment significantly reduced the proportion of cells in G0/G1 phase and increased those in G2/M phase (*p* < 0.05, Figure 1B,C), indicating a shift toward mitotic activity. This pattern aligns with YPFPGPIH’s proliferative role and suggests enhanced cell cycle progression. In support of this, Zeng et al. demonstrated that Rana spinosa meat hydrolysates shorten the DNA synthesis period and accelerate the cell cycle in RAW264.7 cells [31]. Together, these findings suggest that YPFPGPIH promotes macrophage proliferation by modulating cell cycle dynamics. It should be noted that this study did not evaluate potential cytotoxic effects at concentrations above 100 µM or under prolonged exposure conditions.

### 3.2. Enhancing Monocyte Chemotactic Migration with YPFPGPIH

**Figure 2:**
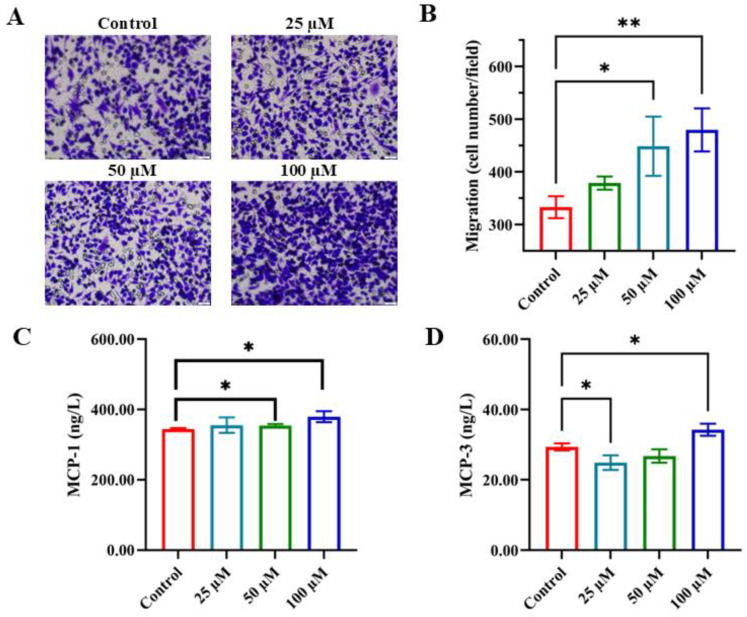
Effects of different concentrations of peptides on the migration of RAW264.7 cells. (**A**) Transwell migration assays showing comparison of migration towards medium and culture supernatants from different concentrations of peptide YPFPGPIH, untreated or treated Raw264.7 cells. Bar = 50 μm. (**B**) Cell migration ability was calculated by counting cells per field. (**C**) The content of MCP-1 in the cell supernatant. (**D**) The content of MCP-3 in the cell supernatant. * *p* < 0.05 compared with the control, ** *p* < 0.01 compared with the control.

Immunomodulation involves the targeted migration and regulation of immune cells—such as T cells, neutrophils, and macrophages—to specific sites like infections, injuries, or tumors. This process is guided by chemotactic gradients, which provide directional cues for immune cell movement [32]. To determine whether YPFPGPIH influences monocyte migration, we performed a Transwell chamber assay using RAW 264.7 macrophages. Culture supernatants pretreated with increasing concentrations of YPFPGPIH significantly enhanced macrophage migration in a dose-dependent manner (Figure 2A,B). Chemokines play a central role in immunomodulation by forming concentration gradients that guide immune cell trafficking [33,34,35]. Specifically, MCP-1/CCL2 and MCP-3/CCL7 promote macrophage migration by binding to the CCR2 receptor [36]. We therefore evaluated the effect of YPFPGPIH on the secretion of these chemokines. As shown in Figure 2C,D, peptide treatment significantly increased the levels of MCP-1 and MCP-3 in the cell supernatant, with the highest concentration (100 µM) inducing the most pronounced effect. This dose-dependent increase aligns with the observed enhancement in cell migration, indicating that YPFPGPIH promotes macrophage migration by upregulating specific chemokines. Cell migration is essential in numerous biological processes, including embryonic development, tissue repair, immune defense, and disease progression [37]. For instance, neural crest cells migrate during embryogenesis to form diverse tissues [38], endothelial cells move toward VEGF signals during angiogenesis [39], neutrophils migrate via CXCL8-CXCR1/2 signaling to infection sites [40], and mesenchymal stem cells homing through the CXCL12-CXCR4 axis aid tissue regeneration [41]. Our findings demonstrate that YPFPGPIH stimulates chemokine secretion in monocytic cells, highlighting its potential as an immunomodulatory agent capable of enhancing immune cell recruitment and fine-tuning inflammatory responses.

### 3.3. The Impact of YPFPGPIH on Cytokine Production

**Figure 3:**
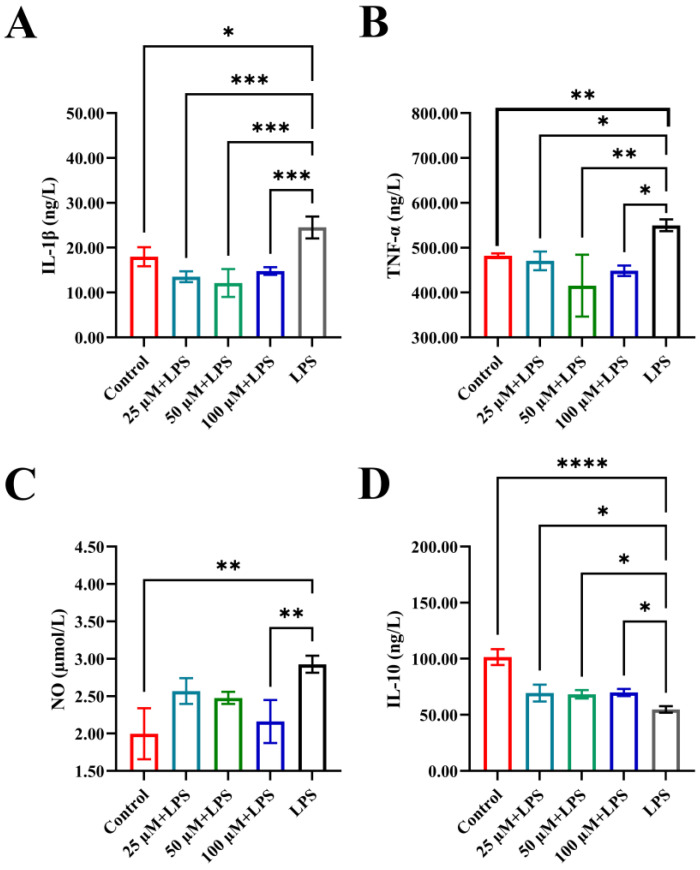
Effects of different concentrations of peptides on inflammatory factors in the mouse macrophage RAW264.7. (**A**) IL-1β, (**B**) TNF-α, (**C**) NO, (**D**) IL-10. * *p* < 0.05 compared with the LPS, ** *p* < 0.01 compared with the LPS, *** *p* < 0.001 compared with the LPS, **** *p* < 0.0001 compared with the LPS.

An imbalance in inflammatory factors can contribute to acute tissue injury or chronic diseases, as these mediators are essential for regulating the body’s response to damage and infection. Dysregulation may result in excessive or insufficient immune activity, underscoring the importance of maintaining inflammatory homeostasis for overall health [42]. To evaluate the immunomodulatory effects of YPFPGPIH, we stimulated RAW264.7 macrophages with LPS and measured the release of IL-10, IL-1β, TNF-α, and NO. IL-1β and TNF-α are key pro-inflammatory cytokines involved in early inflammatory responses, while IL-10 acts as an anti-inflammatory cytokine that suppresses excessive immune activation. As shown in Figure 3, LPS stimulation significantly increased the levels of IL-1β, TNF-α, and NO, while reducing IL-10 secretion, confirming the successful establishment of an inflammatory model. Treatment with YPFPGPIH significantly counteracted these changes in a concentration-dependent manner (25–100 µM). Specifically, the peptide reduced IL-1β and TNF-α levels while increasing IL-10 production, indicating its ability to restore immune balance by modulating both pro- and anti-inflammatory pathways. Notably, at 100 µM, YPFPGPIH also significantly suppressed NO production. Since NO is mainly generated by iNOS, whose expression is regulated by NF-κB [43], this suggests that YPFPGPIH may interfere with iNOS expression or NF-κB activation. Excessive pro-inflammatory mediators can cause systemic inflammation and tissue damage [44]. For instance, TNF-α and IL-17 contribute to rheumatoid arthritis [45], and dysregulated IFN-α promotes lupus erythematosus [46]. While NO helps combat pathogens [47], its overproduction can impair epithelial integrity [48] and suppress anti-tumor immunity [49]. Previous studies have shown that natural compounds such as Phellinus linteus polysaccharides can rebalance inflammatory cytokine networks [50]. Similarly, our results indicate that YPFPGPIH possesses anti-inflammatory properties and enhances immunoregulatory activity in macrophages by modulating IL-10, IL-1β, TNF-α, and NO. These findings collectively demonstrate that YPFPGPIH helps maintain inflammatory homeostasis by fine-tuning the secretion of key immune mediators. Moreover, the observed cytokine profile suggests that YPFPGPHI may modulate macrophage polarization towards an M2-like phenotype, although further investigation using specific polarization markers is required to confirm this hypothesis.

### 3.4. The Impact of YPFPGPIH on Macrophage Phagocytosis

**Figure 4:**
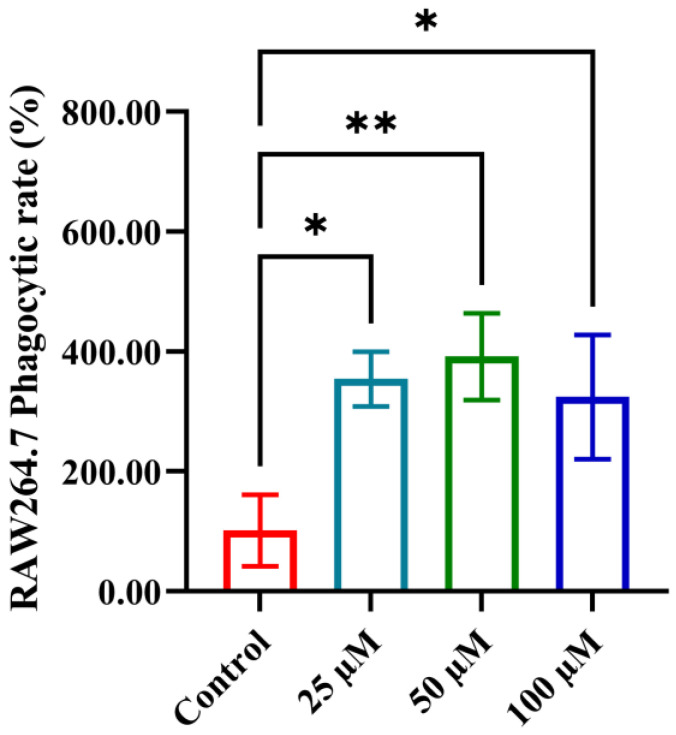
Effects of different concentrations of peptides on phagocytic activity of RAW264.7. * *p* < 0.05 compared with the control, ** *p* < 0.01 compared with the control.

Phagocytosis is a crucial defense mechanism in the immune system, enabling phagocytes to recognize, engulf, and eliminate pathogens and dead cells. This process supports immune regulation and helps initiate adaptive immune responses. We evaluated the effect of YPFPGPIH on the phagocytic activity of RAW264.7 macrophages using a neutral red uptake assay. As shown in Figure 4, increasing concentrations of the peptide significantly enhanced dye uptake (*p* < 0.05), indicating a marked promotion of phagocytosis even at low concentrations. Phagocytosis is a fundamental function of innate immunity, primarily carried out by macrophages and neutrophils [51,52]. Beyond pathogen clearance, it also contributes to antigen presentation, inflammation control, and tissue repair [53,54]. These roles are essential for maintaining host integrity and coordinating immune defense. For instance, Wang et al. reported that the peptide Pep-L2 enhances macrophage phagocytosis of E. coli by binding to the TLR4/MD2 complex, triggering ROS production, and promoting phagosome acidification [55]. Whether YPFPGPIH acts through TLR4 to stimulate phagocytosis via cytokines such as TNF-α or IL-1β remains to be investigated. Since YPFPGPIH significantly promoted macrophage proliferation at 25–100 µM without signs of cytotoxicity, we selected 100 µM for all subsequent experiments to ensure a pronounced biological response.

### 3.5. Impact of YPFPGPIH on Relative mRNA Expression of Immune Factors

**Figure 5:**
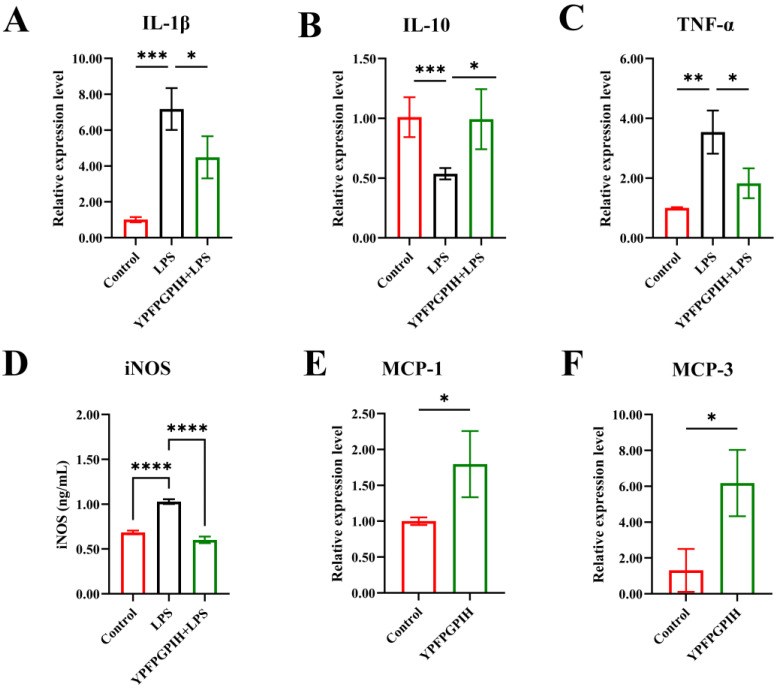
Effects of different peptides on the Relative mRNA expression of inflammatory cytokines and chemokines of RAW264.7 cells. (**A**–**C**) The mRNA relative expression of IL-1β, IL-10, and TNF-α. (**D**) The content of iNOS in the cell. (**E**,**F**) The mRNA relative expression of MCP-1 and MCP-3. * *p* < 0.05, ** *p* < 0.01, *** *p* < 0.001, **** *p* < 0.0001.

The mRNA expression levels of inflammatory cytokines (IL-10, IL-1β, TNF-α), chemokines (MCP-1, MCP-3), and inducible nitric oxide synthase (iNOS) were analyzed by quantitative PCR (Figure 5). Compared to the control group, LPS-stimulated RAW264.7 cells showed significantly increased mRNA expression of IL-1β, TNF-α, and iNOS, along with reduced expression of IL-10. Treatment with YPFPGPIH significantly reversed these changes: it upregulates the mRNA expression of IL-10 while downregulating IL-1β and TNF-α. YPFPGPIH also reduced iNOS mRNA levels. In addition, the peptide enhanced the transcription of the chemokines MCP-1 and MCP-3. Under inflammatory conditions such as LPS exposure, signaling pathways such as NF-κB and MAPK are activated, leading to the transcription and translation of inflammatory mediators. Consistent with this, Long et al. reported that the peptide MAMP-1 suppresses the mRNA expression of IL-6, IL-1β, and TNF-α under inflammation, thereby reducing cytokine release [56]. Our results further indicate that YPFPGPIH modulates inflammatory responses at the transcriptional level. iNOS is highly expressed in immune cells following inflammatory stimulation and contributes to sustained NO production [57]. Our findings show that YPFPGPIH inhibits NO release by downregulating iNOS expression. In parallel, the peptide promotes chemokine production by upregulating MCP-1 and MCP-3 transcription.

### 3.6. Impact of YPFPGPIH on NF-κB and MAPK Signaling Pathways

**Figure 6:**
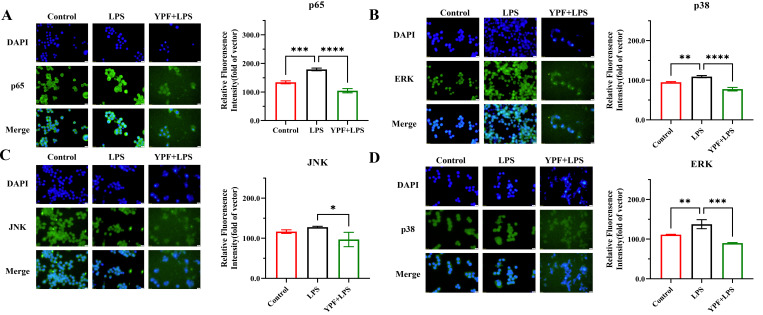
The effect of YPFPGPIH on the immunofluorescence staining and expression levels of NF-κB and MAPK signaling pathway proteins. The left panel shows the protein expression images of p65 (**A**), ERK (**B**), JNK (**C**), and p38 (**D**) observed under a fluorescence microscope; the right panel shows the immunofluorescence analysis of the protein expression levels of p65, ERK, JNK, and p38. Compared with the control group, * *p* < 0.05, ** *p* < 0.01, *** *p* < 0.001, **** *p* < 0.0001.

To investigate the role of NF-κB and MAPK signaling in peptide-mediated immunomodulation, we analyzed key components of these pathways using immunofluorescence staining. As shown in Figure 6, LPS stimulation significantly increased the expression of p65, ERK, JNK, and p38 proteins in RAW264.7 cells compared to the control group (*p* < 0.05). However, treatment with the peptide YPFPGPIH markedly reduced the expression levels of these proteins (*p* < 0.05). The NF-κB and MAPK pathways are central regulators of inflammatory and chemokine gene expression [58]. Upon LPS stimulation, IKK activation induces nuclear translocation of p65, enhancing transcription of inflammatory genes [59]. In the MAPK pathway, ERK promotes IL-6 expression via C/EBPβ or CREB activation; JNK phosphorylates c-Jun to activate TNF-α transcription and cooperates with NF-κB to upregulate IL-1β; and p38 modulates gene expression by phosphorylating ATF2 and CREB, while also stabilizing mRNA through AU-rich elements [60]. Our findings indicate that YPFPGPIH modulates the mRNA expression of inflammatory mediators through the NF-κB and MAPK pathways. By regulating these key signaling networks, YPFPGPIH helps rebalance inflammatory cytokine production, highlighting its potential as a therapeutic agent for inflammatory conditions.

**Figure 7:**
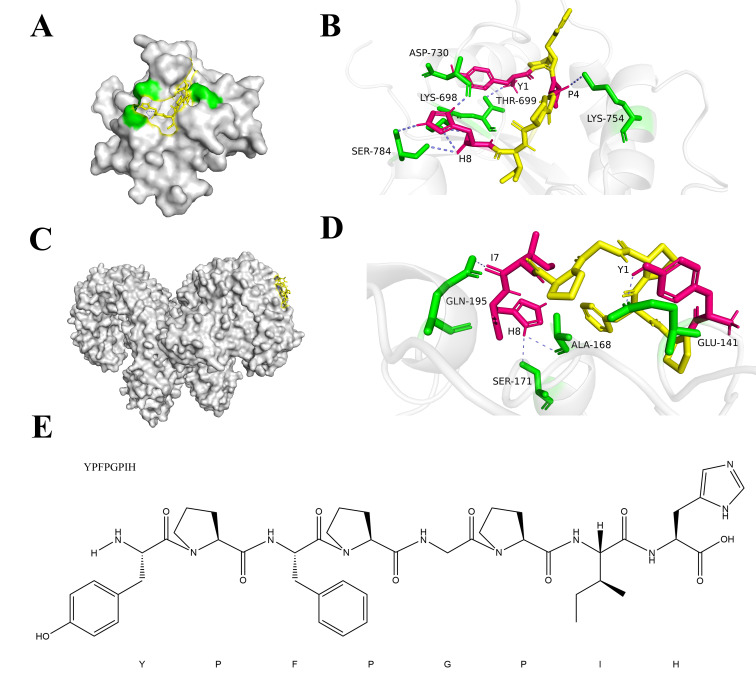
Molecular docking result of YPFPGPIH with TLR2 and TLR4/MyDD: (**A**,**B**) With TLR2. (**C**,**D**) With TLR4/MyDD. (**E**) YPFPGPIH structure.

To investigate the peptide-mediated immunomodulatory mechanisms, we performed molecular docking simulations to assess the interaction between YPFPGPIH and Toll-like receptors (TLRs). The results showed strong binding of YPFPGPIH to both TLR2 and TLR4/MD-2, with binding energies greater than 4 kcal/mol (Table 2). These findings are consistent with previous reports that immunomodulatory peptides typically bind TLRs with energies exceeding 3.5 kcal/mol [61]. Higher binding energy reflects greater complex stability, supporting a structural basis for the immunoregulatory function of YPFPGPIH. Analysis of the binding modes revealed that YPFPGPIH forms six hydrogen bonds with SER784, ASP730, LYS698, THR699, and LYS754 in TLR2, and four hydrogen bonds with GLN195, SER171, ALA168, and GLU141 in TLR4/MD-2 (Figure 7). Notably, tyrosine (Y) and histidine (H) residues in the peptide were critical for hydrogen bonding, aligning with previous studies emphasizing their importance in peptide–immune receptor interactions [62]. These results indicate that YPFPGPIH likely triggers TLR2 and TLR4 signaling through specific binding interactions, rather than via membrane penetration. This engagement is predicted to initiate downstream NF-κB and MAPK cascades, modulating the production of inflammatory mediators. To confirm the specificity and functional relevance of these interactions, future studies should employ TLR2- and TLR4-knockout models, along with biophysical methods such as surface plasmon resonance (SPR), to validate binding affinity and kinetic parameters.

Furthermore, to fully evaluate the potential of YPFPGPIH for functional food development, future investigations should focus on its stability under simulated gastrointestinal conditions and its bioavailability in vivo. It will also be important to examine how YPFPGPIH interacts with other food components and whether its immunomodulatory activity is maintained within complex food matrices. The effects of YPFPGPIH on other immune cells, such as T cells and dendritic cells, remain to be explored to better understand its broader immunoregulatory network. Finally, comprehensive safety assessments—including systematic evaluation of its potential allergenicity and immunogenicity—must be conducted before any practical application of YPFPGPIH can be considered.

## 4. Conclusions

This study demonstrates that the milk-derived peptide YPFPGPIH exhibits multifaceted immunomodulatory activity. Specifically, it enhances key macrophage functions—such as proliferation, phagocytosis, and chemotactic migration—while balancing pro- and anti-inflammatory responses. YPFPGPIH acts through TLR2/4 receptors to suppress NF-κB and MAPK signaling, thereby attenuating excessive inflammatory cytokine release and supporting tissue repair. The peptide also upregulates chemokines MCP-1 and MCP-3, facilitating targeted immune cell recruitment essential for inflammation resolution and immune surveillance. Notably, YPFPGPIH remains effective at low concentrations (25–100 µM), indicating high bioactivity and potential suitability for practical applications. These properties make it a compelling candidate for functional foods aimed at managing chronic inflammation, such as dairy-based supplements. Future studies should focus on validating its in vivo efficacy and further elucidating its systemic immunomodulatory mechanisms.

## Figures and Tables

**Table 1:** Genes and Corresponding Primers Used in RT-qPCR.

Gene	Primer	Sequence (5′ to 3′)
MCP-1	Forward	GGCTCAGCCAGATGCAGTTAA
	Reverse	CCTACTCATTGGGATCATCTTGCT
MCP-3	Forward	AAGAAGGGCATGGAAGTCTG
	Reverse	TCAAGGCTTTGGAGTTGGG
TNF-α	Forward	CTGGATGTCAATCAACAATGGGA
	Reverse	ACTAGGGTGTGAGTGTTTTCTGT
IL-1β	Forward	TGTGAAATGCCACCTTTTGA
	Reverse	TGAGTGATACTGCCTGCCTG
IL-10	Forward	CAGAGCCACATGCTCCTAGA
	Reverse	TGTCCAGCTGGTCCTTTGTT
GAPDH	Forward	AGGTCGGTGTGAACGGATTTG
	Reverse	GCAGCTCTAGGAGCATGTGG

**Table 2:** Molecular Docking of Peptides with Toll Receptors.

Sequence	Affinity (kcal/mol) TLR2	Affinity (kcal/mol) TLR4/MyDD
YPFPGPIH	−9.9	−5.9

## Data Availability

The original contributions presented in this study are included in the article. Further inquiries can be directed to the corresponding author.
